# Is Carpal Tunnel Release Necessary in High-Energy Distal Fractures of the Radius?

**DOI:** 10.7759/cureus.53404

**Published:** 2024-02-01

**Authors:** Muhammed Kazez, Anil Agar, Sefa Key, Orhan Ayas, Mustafa Ümit Gürbüz

**Affiliations:** 1 Department of Orthopedics and Traumatology, Elazıg Fethi Sekin City Hospital, Elazığ, TUR; 2 Department of Orthopedics and Traumatology, Firat University Hospital, Firat University, Elazığ, TUR; 3 Department of Orthopedics and Traumatology, Fırat Universty, Elazığ, TUR; 4 Department of Orthopedics and Traumatology, Elazığ Fethi Sekin Training and Research Hospital, Elazığ, TUR

**Keywords:** plate osteosynthesis, radius distal fracture, high energy, carpal tunnel syndrome, carpal tunnel release

## Abstract

Purpose

This study aimed to compare the clinical outcomes of patients who underwent volar plate osteosynthesis for high-energy distal radius fracture (DRFx) and carpal tunnel release (CTR) for acute or subacute carpal tunnel syndrome (CTS) with patients who did not undergo CTR.

Methods

This study is a retrospective evaluation of all high-energy DRFx treated with volar plate osteosynthesis in a regional hospital between January 2021 and January 2023. All adult patients (≥18 years) who underwent open reduction and internal fixation were included in the study after obtaining approval from the internal review board of our institution. Only patients who underwent plate osteosynthesis of the volar aspect through a modified Henry incision and patients who underwent CTR through a classic separate incision were included in the study. Clinical results include hand dynamometry, visual analog scale (VAS) scores, and physical examination findings of patients who underwent volar plate osteosynthesis because of high-energy DRFx and CTR due to CTS in the acute and subacute periods were retrospectively examined.

Results

Among the patients who underwent volar plate osteosynthesis because of high-energy DRFx, no statistically significant difference was detected between the hand grip strength and VAS scores of patients who underwent CTR because of acute CTS and subacute CTS at the sixth postoperative week (p>0.05).

Conclusion

Prophylactic CTR may be performed in the same session in selected cases, such as DRFx caused by a high-energy injury, to establish a scale for DRFx at a high risk of CTS and avoid delays in treatment. CTR for transient CTS detected in the subacute period during outpatient follow-up does not improve clinical outcomes.

## Introduction

The most common fractures seen in emergency departments are distal radius fractures (DRFx), which account for approximately 3% of all upper extremity injuries and have an annual incidence of more than 640,000 in the United States alone. Arthrosis, malunion nonunion, tendon rupture, chronic regional pain syndrome, ulnar impaction syndrome, loss of rotation, finger stiffness, and compartment syndrome are common complications after DRFx [1]. One of the most important complications of DRFx is carpal tunnel syndrome (CTS) [2,3], the incidence of which is estimated to vary between 3.3% and 17.2% [4,5].

Following DRFx, CTS is an important complication that can present with acute, transient, or delayed symptoms. Acute CTS occurs immediately after injury, whereas transient CTS in the subacute phase occurs weeks or even months late. Delayed-onset CTS may develop years after the initial injury [6]. Acute CTS, which has an incidence of 5.4% to 8.6% after DRFx, is characterized by worsening severe pain and paresthesia occurring hours after a fracture in the median nerve distribution of the hand. The cause is thought to be increased compartment pressure in the carpal tunnel [7,8]. Transient CTS, which has an estimated incidence of 4% and is the least understood of the three etiologies, is most likely caused by nerve stretching or contusion [9]. Unlike acute CTS, transient CTS symptoms may be present at the time of injury but may not traditionally progress; instead, they may get progressively better over days and weeks [9,10]. Finally, delayed CTS, which can develop months or years after an accident and has an incidence of 0.5% to 22% following DRFx, is typically caused by a change in the structure of the carpal tunnel after the fracture has healed [4].

In the case of a high-energy mechanism or a related fracture pattern, carpal tunnel release (CTR) may be performed as treatment or prophylactic depending on the severity of the symptoms [6]. Open reduction and internal fixation of the DRFx can be performed at the same time as CTR; however, in some cases, CTS may occur postoperatively and require subsequent CTR. For the best outcomes, acute CTS has been shown to require early diagnosis and care [11,12].

The aim of this study was to compare the clinical outcomes of patients who developed CTS after volar plate osteosynthesis for high-energy DRFx.

## Materials and methods

**Figure 1:**
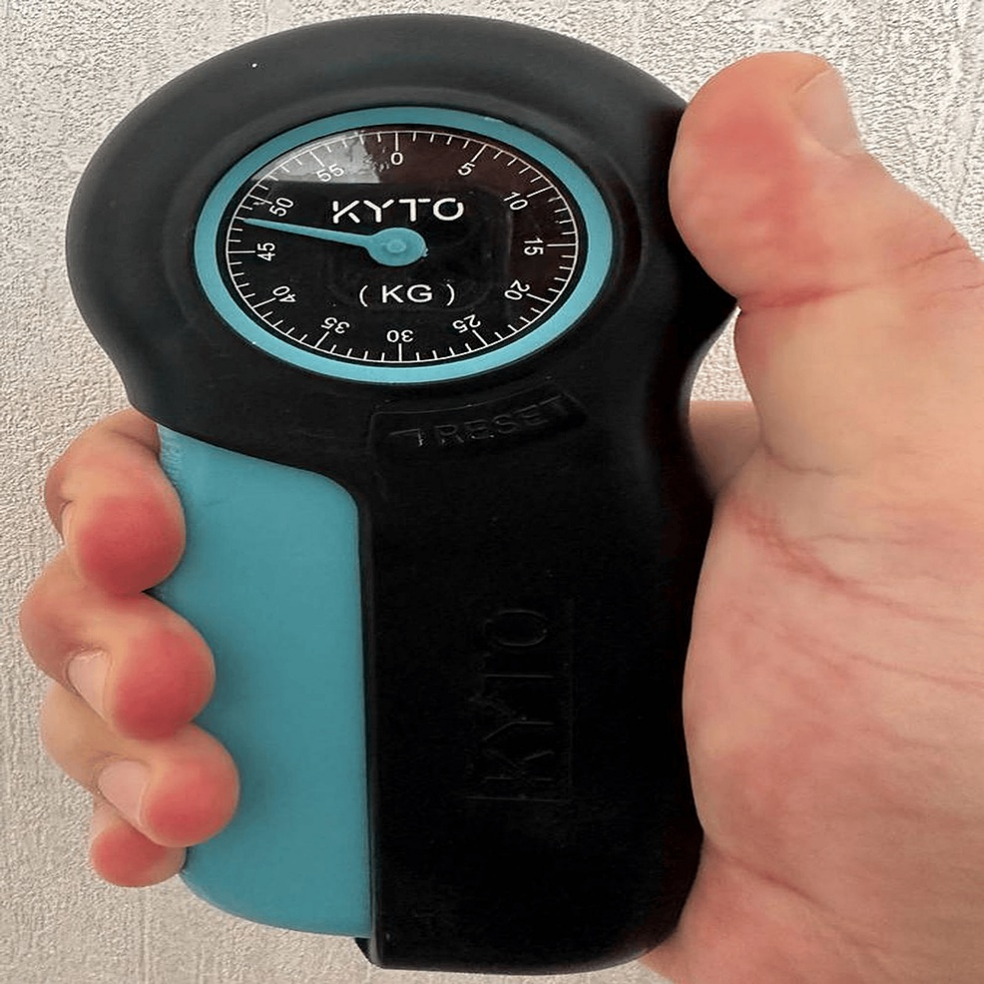
Hand dynamometer

This study is a retrospective evaluation of all high-energy DRFx treated with volar plate osteosynthesis in a regional hospital between January 2021 and January 2023. Approval for the study was obtained from the Firat University Non-Interventional Research Ethics Committee (03.08.2023-17486). Demographic characteristics of the patients, follow-up periods, whether CTR was performed because of acute or subacute CTS in patients operated for DRFx, presence of typical carpal tunnel physical examination findings, presence of additional fractures, and the etiology of the trauma causing DRFx were evaluated. Hand grip strength was measured with a KYTO Hand Dynamometer (Model EH101, China), with a grip strength meter hand force power of 121LB/55kg) at postoperative week 12 (Figure 1).

An electromyography (EMG) study was performed in the sixth postoperative week. The distal motor delay was measured by stimulating the median nerve in the distal forearm, 7 cm proximal to the recording electrode centered on the thenar muscle. A delay longer than 4.2 milliseconds was considered long-term (positive/+) [13,14].

All wrists were immobilized with a volar splint for approximately two weeks postoperatively, and active and passive finger movement exercises were started on the second postoperative day. Early active movement of the wrist was achieved starting from the second postoperative week. Visual analog scale (VAS) score was measured at postoperative week 12 to evaluate the differences between the groups.

Patients were followed up by the same orthopedic specialist team. The following criteria were used for the diagnosis of CTS: (1) positive EMG test; (2) positive Durkan or Phalen test at the wrist; and (3) numbness, pain, and sensory loss in the median nerve sensory area. As the abovementioned physical examination findings and EMG tests could not be performed for the diagnosis in patients who developed acute CTS because of DRFx, the following criteria were used for the diagnosis of CTS: severe pain in the hand and wrist, numbness in the first, second, and third finger and loss of sensation in the median nerve sensory area.

The patients were followed by the same orthopedic specialist team. The following criteria were used for surgical indication in the diagnosis of CTS: (1) positive EMG test; (2) positive Durkan or Phalen test; and (3) numbness, pain, and sensory loss in the median nerve sensory area. The above criteria could not be used for the diagnosis of CTS in the acute phase of DRFx, and severe pain, numbness, and sensory loss in the median nerve sensory area were accepted as diagnostic criteria for the diagnosis of CTS in these patients.

Inclusion and Exclusion Criteria

The study included patients aged 18 years and older, patients with DRFx because of high-energy trauma, patients who underwent modified Henry incision from the volar side, patients who underwent open reduction, patients who underwent the same type and brand of plate osteosynthesis, and patients who had anatomical reduction and adequate joint stabilization during fracture fixation. Among the patients who underwent CTR because of CTS, only patients who underwent CTR through a classical separate incision were included in the study. Patients with inadequate follow-up, patients under 18 years of age, patients previously operated for ipsilateral DRFx, patients with a history of ipsilateral chronic CTS, and patients who underwent previous CTR were excluded.

Surgical Technique

**Figure 2:**
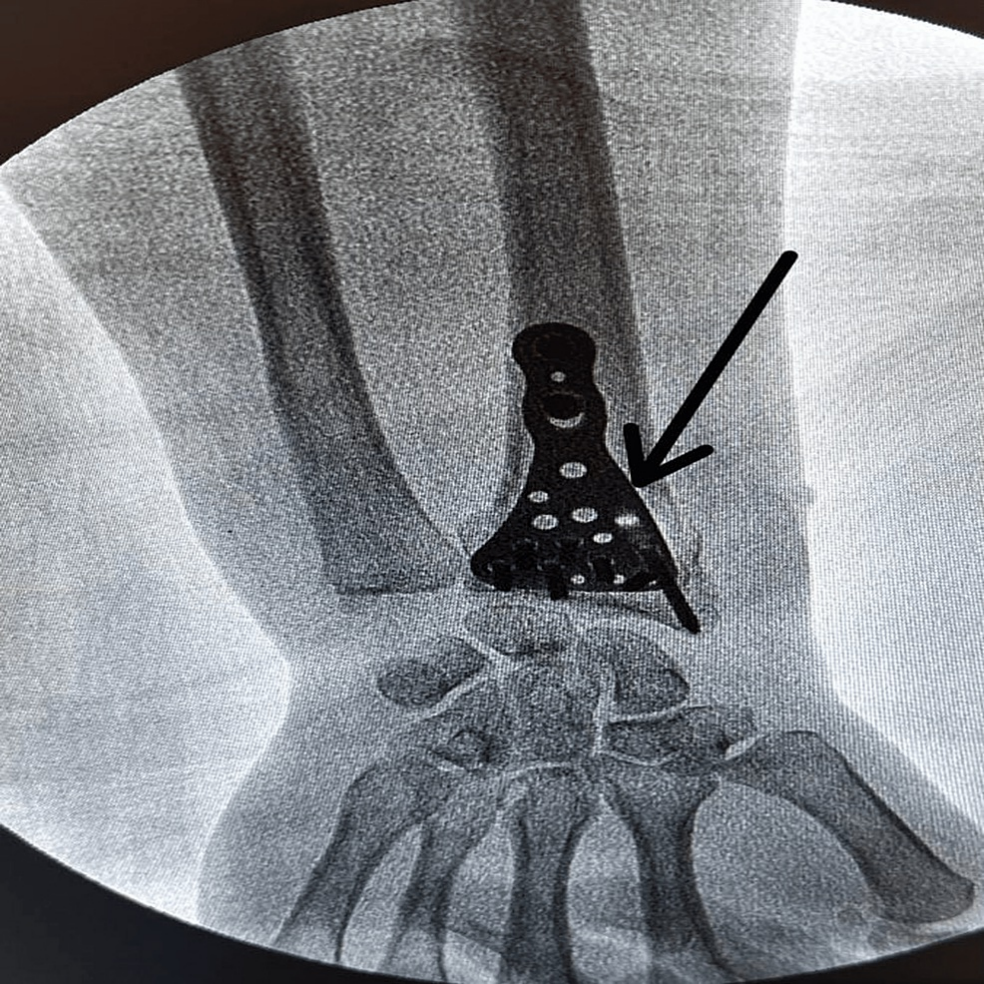
Osteosynthesis with a volar-locked radius distal anatomical plate

The surgery was performed via the volar approach. A longitudinal incision was made on the flexor carpi radialis tendon. The flexor tendon sheath was opened longitudinally to allow retraction of the tendon in the ulnar direction. The flexor policis longus and flexor carpi radialis tendons were medialized, and the radial artery was lateralized and entered through. The pronator quadratus was stripped from the radius in an L-shape. After anatomical reduction of the joint surface and ensuring the appropriate alignment and length for the bone pieces in the metaphyseal region, osteosynthesis was completed with a volar-locked radius distal anatomical plate (TST, Istanbul) with the help of fluoroscopy (Figure 2).

**Figure 3:**
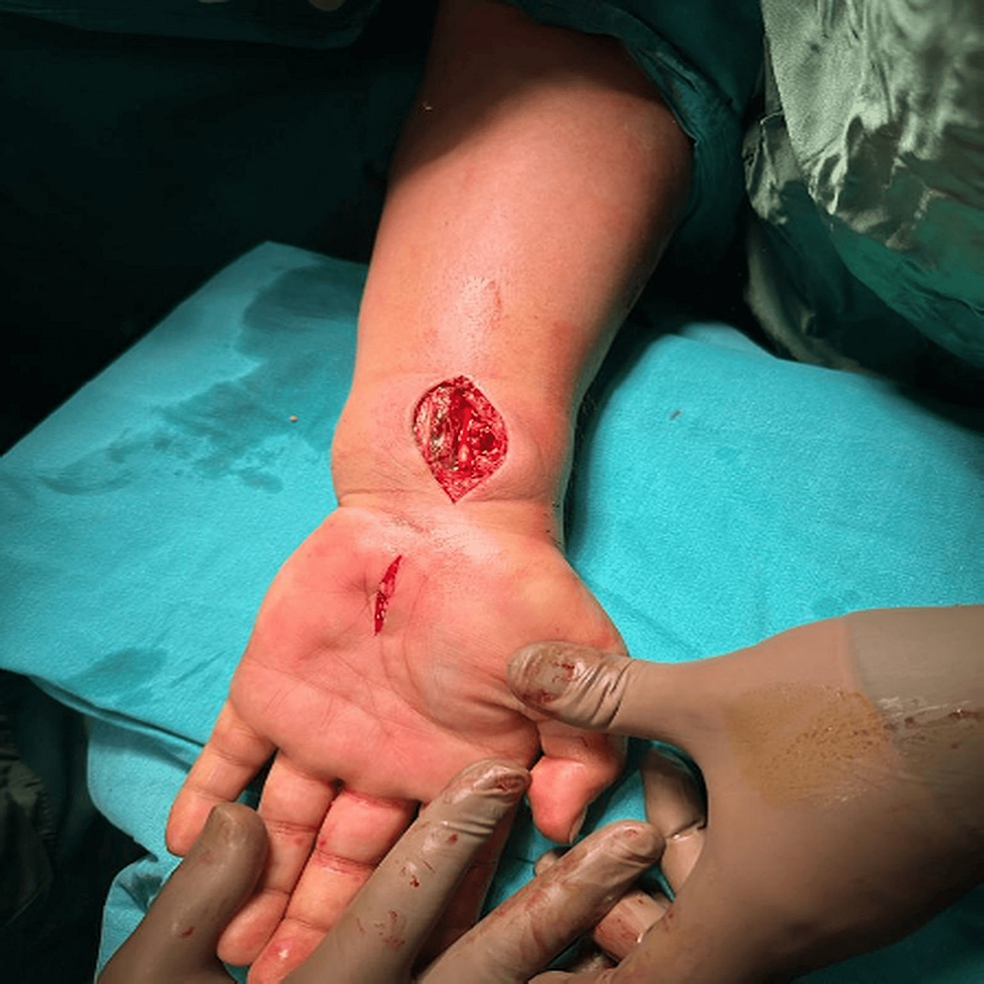
Classic separate incision for CTS

In all patients who underwent CTR, the release was performed through a classic separate skin incision (Figure 3). The incision was made at the intersection of Kaplan's cardinal line and the third web space line, and it was completed with full-length visualization of the transverse carpal ligament and removal of pressure on the median nerve. Then only the skin was sutured.

Statistical Analysis

The Statistical Product and Service Solutions (SPSS, version 22; IBM SPSS Statistics for Windows, Armonk NY) program was used for statistical analyses while evaluating the findings obtained in the study. While evaluating the study data, the conformity of the parameters to normal distribution was evaluated by Shapiro Wilks test. In addition to descriptive statistical methods (mean, standard deviation, frequency), Mann-Whitney U test was used for comparisons of quantitative data between two groups for parameters that did not show normal distribution. Significance was evaluated at p<0.05 level.

## Results

After the exclusion criteria were applied, 54 patients were included in the study. Of these patients, 45 (83.3%) were male and nine (16.7%) were female. The ages of the patients ranged between 18 and 63 years with a mean of 38.09±10.72 years. Follow-up period values ranged between 121 and 380 days with a mean of 151.11±15.51 days.

While 74% of the patients did not undergo CTR (Group 1), 22.2% underwent CTR simultaneously with surgery for acute CTS (Group 2), and 3.8% underwent CTR for CTS in the subacute period (Group 3). Two patients were diagnosed with CTS in the subacute period, but these patients refused surgery (Group 4). Physical examination findings in the sixth week of the postoperative period showed that 35.2% of the patients had complaints of numbness, pain, or loss of sensation in the hand, while 64.8% of the patients did not have these complaints. Although 35.2% of the patients had complaints of numbness, pain, or loss of sensation in their hands, EMG tests of these patients showed positive values for CTS in 7.4% of the patients and negative values for CTS in 92.6% of the patients.

Additional trauma: Open fracture in 14.8%, bilateral DRFx in 20.3%, carpal bone fracture in 7.4%, distal humerus fracture in 11.1%, proximal humerus fracture in 1.9%, soft tissue injury in 31.5%, and other system injuries in 13%. When the etiologies of trauma were analyzed, it was seen that 16.7% of the patients were injured because of work accidents, 1.9% because of traffic accidents, and 81.4% because of falling from a height.

When the hand grip strength of the patients was evaluated, no statistically significant difference was found between the groups, wherein CTR was performed because of CTS (Group 2 and Group 3) and the groups wherein CTR was not performed (Group 1 and Group 4) in terms of hand grip strength test values (p=0>0.05) (Table 1).

**Table 1:** Evaluation of hand grip strength test values according to groups

		Hand grip strength test		
		N	Min-Max	Mean±SD (median)
Patients	Group 1	40	25-50	42.63±5.93 (45)
	Group 2	12	25-50	41.67±8.07 (45)
	Group 3	2	35-40	37.5±3.54 (37.5)
	p			0.955
Patients	Group 3	2	35-40	37.5±3.54 (37.5)
	Group 4	2	36-40	38±2.83 (38)
	p			0.648

When the postoperative VAS scores of the patients were evaluated, there was no significant difference between the groups (p=0.981>0.05) (Table 2).

**Table 2:** Evaluation of VAS scores according to groups

		VAS score last follow-up		
		N	Min-Max	Mean±SD(median)
Group	Group 1	40	1-4	2.45±0.68 (2)
	Group 2	12	1-4	2.42±0.9 (2.5)
	Group 3	2	2-3	2.5±0.71 (2.5)
	Total	54	1-4	2.44±0.72 (2)
	p			0.981

## Discussion

This study aimed to compare the clinical outcomes of patients who developed CTS after volar plate osteosynthesis for high-energy DRFx. Although there are similar studies in the literature on the development of CTS in DRFx, our study investigated the development of CTS in a specific group of patients such as high-energy DRFx. The higher incidence of acute CTS in DRFx caused by high-energy injuries in our study compared to the literature is important and requires attention. In our study, we found that the clinical outcomes of patients who underwent CTR because of CTS in the subacute period were like those of patients who complained of numbness in their hands in the subacute period but whose CTS diagnosis could not be confirmed by EMG testing and, hence, did not undergo CTR.

Prophylactic CTR may be useful after surgical treatment of DRFx to prevent early or late onset of median nerve dysfunction. Consideration should be made for certain fracture patterns, such as significant displacement, comminution, or concomitant radiocarpal dislocation, which predispose to the development of acute CTS [15,16]. Fractures requiring repeated closed reduction attempts and fractures immobilized in high flexion are additional risk factors for median nerve impairment [16]. As the DRFx of the patients included in our study initially met the criteria of multipartite and instability, closed reduction and plaster treatment were not attempted, and temporary fixation was made with a short-arm splint until the operation. In other cases where patient-related factors may mask the occurrence or development of median nerve dysfunction, prophylactic CTR during fracture repair may be useful. Within hours after the onset of nerve compression, animal models have demonstrated persistent peripheral nerve dysfunction or nerve damage caused by various biological reactions such as endoneurial edema, demyelination, inflammation, distal axonal degeneration, and fibrosis [17].

Patients who have undergone several surgeries, suffered a head injury, or are intubated may experience postoperative median nerve entrapment that goes unnoticed because they are unable to communicate their postoperative sensory deficiencies [18]. Furthermore, depending on the anesthetic agent used, patients may be unable to consistently identify sensory or motor deficits unrelated to the anesthesia up to 24 hours following surgery because of the rise in the use of regional anesthetic for outpatient upper extremity surgery. After fixing the DRFx, preventative CTR may help address these patients' concerns about postoperative compressive median neuropathy and delay in diagnosis [18]. 

A typical side effect of DRFx is delayed median neuropathy. Bienek et al. found delayed CTS confirmed by electrodiagnostic testing in 12 of 60 patients (20%) an average of 10 months after DRFx [19]. In a retrospective review of 49 surgically repaired DRFx, Jupiter et al. reported three cases of delayed CTS (6%), all treated because of CTR [20]. In a later retrospective analysis by Ruch and Papadonikolakis, two of 16 surgically repaired DRFx required additional surgery for delayed CTS (12.5%) [21]. In a study by Arora et al., delayed CTS was seen in three (3%) of 114 patients who underwent volar plating for DRFx. All of these patients required additional surgery for the removal of the CTS and hardware [22]. We think that the 22% higher rate of acute CTS in this study compared to the literature is because the study included only high-energy DRFx. As surgical treatment was performed in all patients who developed acute CTS in our study, it is not known what kind of CTS complications would have occurred if prophylactic CTR had not been applied in this series. Consistent with the literature, no significant difference was observed in grip strength and VAS scores in patients with acute CTS compared to other patients in this study. Evidence-based studies are needed on the approach to CTS intervention, preoperative, postoperative, and indication for CTS prophylaxis for DRFx [23]. Caution should be exercised when approaching prophylactic carpal tunnel surgery. The results of our study do not support the need for prophylactic carpal tunnel surgery. According to Niver et al., CTR is better managed after the complete healing of the DRFx. There is no role for prophylactic CTS during distal radius fixation in an asymptomatic patient [8]. However, 31.56% of patients with electromyographic findings at six months were found to have CTS without CTS symptoms [24]. In our study, although 35,2 % of the patients had typical CTS physical examination findings such as numbness and pain in the hands, only 7.4% of these patients had a positive EMG test. In our study, CTS criteria based on both EMG examination and clinical symptoms were accepted as indications for CTR. Comparison of VAS scores at six months postoperative in patients with and without prophylactic CTR after DRFx showed less pain in the CTR group [24]. In our study, no significant difference was observed in VAS scores after concomitant carpal tunnel surgery for high-energy DRFx. Furthermore, when hand grip strength was evaluated, there was no statistically significant difference in strength test scores between the non-release and surgical groups.

As there were no patients in our study who did not undergo release because of acute CTS, no comparison could be made between the acute groups. Additionally, altered anatomy, cross-sectional area, and compartment pressure of the carpal tunnel because of misunion of the distal radius may lead to CTS. This altered anatomy is thought to cause median nerve compression and/or traction mechanism. This may predispose the individual to either the development of new-onset CTS or exacerbation of preexisting subclinical median nerve compression neuropathy [25]. Similarly, although improvements in VAS scores were observed in our study, no significant difference was found between the two groups. Grip strength has been used as one of the indicators of functional recovery after CTR [26]. There are numerous reports on grip strength recovery patterns after CTR, and most researchers suggest that patients usually reach full grip strength recovery within three to six months postoperatively [27]. Some investigators have reported a rapid improvement in grip strength within four to six weeks postoperatively [28]. In our study, the functional results of the wrist were evaluated according to the hand grip strength with a wrist dynamometer at 12 weeks postoperatively. Possible unfavorable anatomical changes in the wrist in CTS after DRFx should be considered. Another study reported more cases of CTS in the prophylactic CTR group because different approaches were used to release the carpal tunnel [29]. In our study, as the same approach was used in all patients undergoing CTR, no comparison could be made in this regard.

It has been reported that factors such as additional medical problems and fracture type should be considered for guideline purposes to confirm the need for carpal surgery in DRFx, and further studies are needed in this regard [30]. The higher rate of acute CTS in our study compared to the literature suggests that the fracture mechanism and high-energy injuries cause a lot of edema in and around the wrist, increasing the risk of CTS.

Limitations and Strength

The fact that we only observed a specific group of patients with high-energy DRFx is one of the strengths of our study. The use of EMG testing in the diagnosis of CTS increased the diagnostic reliability in terms of qualitative measurement of patients who were diagnosed based only on physical examination findings, and this is another strength of the study. Limitations of the study include the short follow-up period, wide age range, and the presence of additional fractures because of a high-energy injury. Additionally, the inability to use the EMG test in the diagnosis of acute CTS and making the diagnosis based only on physical examination findings are also limitations of the study. Another limitation of the study is that we did not have any patients who did not undergo relaxation because of acute CTS, and these patients could not be compared with other patients.

## Conclusions

There is not a high level of evidence supporting prophylactic CTR for DRFx. In selected cases such as DRFx caused by a high-energy injury, the incidence of acute CTS is increased, and caution should be exercised. Prophylactic CTR can be performed in the same session to prevent delays in treatment for DRFx caused by high-energy injury with a high risk of CTS. For transient CTS detected in the subacute period during outpatient follow-up, CTR does not improve clinical outcomes. In the future, a consensus may be reached as the number of studies in this field increases and follow-up periods are extended.

## References

[1] Chung KC, Spilson SV (2001). The frequency and epidemiology of hand and forearm fractures in the United States. J Hand Surg Am.

[2] McEntee RM, Tulipan J, Beredjiklian PK (2023). Risk factors and outcomes in carpal tunnel syndrome following distal radius open reduction internal fixation. J Hand Surg Am.

[3] Kim KH, Duell B, Munnangi S, Long M, Morrison E (2022). Radiographic predictors of delayed carpal tunnel syndrome after distal radius fracture in the elderly. Hand (N Y).

[4] Cooke ME, Gu A, Wessel LE, Koo A, Osei DA, Fufa DT (2022). Incidence of carpal tunnel syndrome after distal radius fracture. J Hand Surg Glob Online.

[5] Rothman A, Samineni AV, Sing DC, Zhang JY, Stein AB (2023). Carpal tunnel release performed during distal radius fracture surgery. J Wrist Surg.

[6] Itsubo T, Hayashi M, Uchiyama S, Hirachi K, Minami A, Kato H (2010). Differential onset patterns and causes of carpal tunnel syndrome after distal radius fracture: a retrospective study of 105 wrists. J Orthop Sci.

[7] Dyer G, Lozano-Calderon S, Gannon C, Baratz M, Ring D (2008). Predictors of acute carpal tunnel syndrome associated with fracture of the distal radius. J Hand Surg Am.

[8] Niver GE, Ilyas AM (2012). Carpal tunnel syndrome after distal radius fracture. Orthop Clin North Am.

[9] Pope D, Tang P (2018). Carpal tunnel syndrome and distal radius fractures. Hand Clin.

[10] Kinley DL, Evarts CM (1968). Carpal tunnel syndrome due to a small displaced fragment of bone. Report of a case. Cleve Clin Q.

[11] Ku YC, Gannon M, Fang W, Norcini RC, Woodberry KM (2023). Management of acute carpal tunnel syndrome: a systematic review. J Hand Surg Glob Online.

[12] Mathews AL, Chung KC (2015). Management of complications of distal radius fractures. Hand Clin.

[13] Nakamichi K, Tachibana S (1993). Unilateral carpal tunnel syndrome and space-occupying lesions. J Hand Surg Br.

[14] Alanazy MH (2017). Clinical and electrophysiological evaluation of carpal tunnel syndrome: approach and pitfalls. Neurosciences (Riyadh).

[15] Odumala O, Ayekoloye C, Packer G (2001). Prophylactic carpal tunnel decompression during buttress plating of the distal radius--is it justified?. Injury. Injury.

[16] Handoll HHG, Madhok R (2003). Closed reduction methods for treating distal radial fractures in adults. Cochrane Database Syst Rev.

[17] Rempel D, Dahlin L, Lundborg G (1999). Pathophysiology of nerve compression syndromes: response of peripheral nerves to loading. J Bone Joint Surg Am.

[18] Kent CD, Bollag L (2010). Neurological adverse events following regional anesthesia administration. Local Reg Anesth.

[19] Bienek T, Kusz D, Cielinski L (2006). Peripheral nerve compression neuropathy after fractures of the distal radius. J Hand Surg Br.

[20] Jupiter JB, Fernandez DL, Toh CL, Fellman T, Ring D (1996). Operative treatment of volar intra-articular fractures of the distal end of the radius. J Bone Joint Surg Am.

[21] Ruch DS, Papadonikolakis A (2006). Volar versus dorsal plating in the management of intra-articular distal radius fractures. J Hand Surg Am.

[22] Arora R, Lutz M, Hennerbichler A, Krappinger D, Espen D, Gabl M (2007). Complications following internal fixation of unstable distal radius fracture with a palmar locking-plate. J Orthop Trauma.

[23] Al-Amin Z, Senyürek SA, Van Lieshout EM, Wijffels MM (2018). Systematic review and pooled analysis of the rate of carpal tunnel syndrome after prophylactic carpal tunnel release in patients with a distal radius fracture. Hand Surg Rehabil.

[24] Medici A, Meccariello L, Rollo G, De Nigris G, Mccabe SJ, Grubor P, Falzarano G (2017). Does routine carpal tunnel release during fixation of distal radius fractures improve outcomes?. Injury.

[25] Kwasny O, Fuchs M, Schabus R (1994). Opening wedge osteotomy for malunion of the distal radius with neuropathy: 13 cases followed for 6 (1-11) years. Acta Orthop Scand.

[26] Lee SH, Gong HS (2022). Grip strength measurement for outcome assessment in common hand surgeries. Clin Orthop Surg.

[27] Gutiérrez-Monclus RG, Gutiérrez-Espinoza HJ, Flores-Astudillo AR, Lluch-Homedes AL, Aguirre-Jerez M (2018). Release with or without reconstruction of the transverse carpal ligament for severe carpal tunnel syndrome: a randomized clinical trial. J Hand Surg Eur Vol.

[28] Bai J, Kong L, Zhao H, Yu K, Zhang B, Zhang J, Tian D (2018). Carpal tunnel release with a new mini-incision approach versus a conventional approach, a retrospective cohort study. Int J Surg.

[29] Jagadish U, Ethiraj P, Umesh M, Arun HS (2023). Incidence of carpal tunnel syndrome in distal radius fractures treated by various modalities in a tertiary care center: a single center study. Cureus.

[30] Tulipan JE, Ilyas AM (2020). Carpal tunnel syndrome surgery: what you should know. Plast Reconstr Surg Glob Open.

